# Adverse obstetric and perinatal outcomes in 2333 singleton pregnancies conceived after different endometrial preparation protocols: a retrospective study in China

**DOI:** 10.1186/s12884-022-04682-3

**Published:** 2022-05-01

**Authors:** Zexin Yang, Xuelian Bai, Ying Han, Zhangxiang Zou, Yazhen Fan, Xinyan Wang, Haining Luo, Yunshan Zhang

**Affiliations:** 1 Tianjin Medical University, Tianjin, 300070 China; 2 Tianjin Central Hospital of Gynecology Obstetrics/Tianjin Key Laboratory of Human Development and Reproductive Regulation, Tianjin, 300100 China

**Keywords:** Frozen embryo transfer (FET), Obstetric outcome, Perinatal outcome, Programmed cycle, Natural cycle

## Abstract

**Backgroup:**

Frozen-thawed embryo transfer is rising worldwide. One adverse effect of programmed frozen embryo transfer (FET) reported in some studies is an increased risk of adverse obstetric and perinatal outcomes. Meanwhile, body mass index (BMI) also has adverse effect on obstetric and perinatal outcomes. In this study, we investigated that the influence of different endometrial preparation protocols on obstetric and perinatal outcomes and the role of BMI in it.

**Method:**

This retrospective cohort study included 2333 singleton deliveries after frozen-thaw embryo transfer at our centre between 2014 and 2021, including 550 cycles with programmed FET, 1783 cycles with true natural cycle FET (tNC-FET). In further analysis according to BMI grouped by Asian criterion, group A (18.5 kg/m^2^ ≤ BMI < 24.00 kg/m^2^) included 1257 subjects, group B (24 kg/m^2^ ≤ BMI < 28.00 kg/m^2^) included 503 subjects and group C (BMI ≥ 28 kg/m^2^) included 573 subjects. Baseline characteristics of the two groups were compared and analyzed. Binary logistic regression analyses were performed to explore the association between obstetric and perinatal outcomes and endometrial preparation protocols.

**Results:**

There were no significant differences in the placenta previa, gestational diabetes mellitus(GDM), preterm premature rupture of membranes (PPROM), cesarean section (CS) and macrosomia between the tNC-FET and programmed FET groups (*P* > 0.05). The programmed FET cycles were associated to a higher risk of pregnancy-induced hypertension (PIH) compared with the tNC-FET cycles (7.3% vs 4.4%, crude OR 1.71[1.16–2.54]; adjusted OR 1.845[1.03–3.30]). After dividing the patients into three groups according to the BMI, The programmed FET cycles were associated to a higher risk of PIH in group C (14.4% vs 6.2%, crude OR 2.55 [1.42–4.55]; adjusted OR 4.71 [1.77–12.55]) compared with the tNC-FET cycles. But there was no statistically significant difference in group A and group B. Programmed FET group compared with the tNC-FET group, the risk of PIH increase as the body mass index increase.

**Conclusion:**

This study showed a tendency toward increasing risk of PIH in programmed FET cycle compared with the tNC-FET cycle, and the risk of PIH increases as BMI increases. Increased risk of preterm birth and low birth weight is linked to increased risk of PIH.

**Supplementary Information:**

The online version contains supplementary material available at 10.1186/s12884-022-04682-3.

## Introduction

The use of frozen-thawed embryo transfer (FET) has increased over the past decade with improvements associated with vitrification compared with older slow-freeze methods [1]. This strategy facilitates elective single-embryo transfer (e-SET), reduces the risk of ovarian hyperstimulation syndrome (OHSS), and allows time for results from preimplantation genetic testing (PGT) to return [2–4]. Moreover, potential benefits of FET include a decrease in the incidence of low birth weight, small for gestational age (SGA), preterm birth, placenta previa, placental abruption, and perinatal mortality compared with fresh embryo transfer [5].

However, the latest observational data assessing neonatal and maternal outcomes after FET have suggested higher rates of hypertensive disorders of pregnancy (HPD), postpartum hemorrhage, post-term birth, and macrosomia specifically in programmed FET cycles compared with natural and stimulated cycles [3, 6–8].

There are three main FET protocols used in the clinic, including true natural cycle FET (NC-FET), modified natural cycle FET (mNC-FET) and programmed cycle FET (programmed FET). Cryopreserved embryos must be transferred to the uterus during the critical endometrial window to establish pregnancy [9]. For women with regular menstrual cycles, they can choose to perform in a natural cycle FET (NC-FET), whether it is a tNC-FET or a mNC-FET. In tNC-FET protocol, the time of transplantation was chosen follow the natural menstrual cycle of patients. In mNC-FET protocol, ovulation is triggered by the injection of human chorionic gonadotropin (hCG) instead of a spontaneous LH surge. For women who have irregular menstrual cycles or for some logistical reasons, a programmed cycle FET (programmed FET) initiated by estradiol and progesterone is a common choice. In programmed FET protocol, the ovary is suppressed using exogenous estrogen and progesterone, and thus there is no development of a dominant follicle. Ovulation does not occur and there is no corpus luteum (CL). The time of transfer is based on the number of days that have passed since the start of exogenous progesterone.

Adverse obstetric outcomes after FET may partly be explained by the FET protocol [10]. Obstetric and perinatal outcomes after the use of different FET protocols have previously been compared. An increased risk of HDP, preterm prelabor rupture of membranes (PPROM), bleeding disorders, cesarean section (CS), post-term birth, macrosomia, and placenta accreta after programmed FET [11, 12] is observed. CL may play an important role in these in the occurrence of these complications. Recent review studies have shown that in pregnant women without CL, the increased risk of pre-eclampsia and impaired maternal vascular health may be due to insufficient secretion of vasoactive substances, such as relaxin, vascular endothelial growth factor and so on [6].

The purpose of our study was to investigate the obstetric and perinatal outcomes of singleton deliveries after using different FET protocols in the Chinese national cohort.

## Materials and methods

### Study design

This retrospective cohort study was conducted at the Center for Reproductive Medicine, Tianjin Central Obstetrics and Gynecology Hospital from January 1, 2014, to June 30, 2021, via our electronic records. The inclusion criteria were as follows: 1) age ≤ 40 years at the time of commencement of in vitro fertilization/intracytoplasmic sperm injection (IVF/ICSI) treatment; 2) singleton pregnancies conceived by FET after IVF/ICSI; The following exclusion criteria were used: 1) oocyte donor treatment cycles; 2) presence of chromosomal abnormalities (included chromosome polymorphism); 3) history of uterine surgery(included endometrial polyps, cesarean and so on); 4) presence of intracavitary lesions, such as endometrial polyps; 5) preimplantation genetic testing; 6)slow freeze cycle. Frozen embryo transfer treatments were grouped according to the stimulation protocol used in the FET cycle, the two FET groups were defined as follows: 1) Programmed FET cycle: women who received no hCG during the FET cycle. All women were treated with progesterone and/or estradiol with or without prior downregulation with GnRH agonist/antagonist.

2) tNC-FET cycle: no medication during FET cycle. We determined and grouped body mass index (BMI) as kilograms per square meter using Chinese criteria [13]. We separated the participants into three groups including the normal-weight group (group A: 18.5 kg/m^2^ ≤ BMI < 24.00 kg/m^2^), overweight group (group B: 24 kg/m^2^ ≤ BMI < 28.00 kg/m^2^), and obesity group (group C: BMI ≥ 28 kg/m^2^). All patients underwent COS according to the routine protocol of our center. Patients received luteal support after embryo transfer in the form of intramuscular progesterone 80 mg/day or intravaginal progesterone gel 90 mg/day.

The outcomes measured in our retrospective study were obstetric complications, including preterm birth (PTB), PPROM, PIH (including pregnancy-induced hypertension, preeclampsia, and eclampsia), placenta previa, gestational diabetes mellitus (GDM), CS, birth weight (low birth weight and macrosomia infant). PTB was defined as delivery of live birth < 37 weeks of gestation. PPROM was defined as membranes rupture < 37 weeks of gestation. Low-birth-weight infant was defined as birth weight <2500 g and macrosomia was defined as birth weight >4000 g. In all groups gestational age was calculated based on a first-trimester ultrasonography scan. All diagnoses were allocated by same medical doctors. All diagnoses were allocated by medical doctors.

### Statistical analysis

Statistical analysis was performed with the use of SPSS25.0. Student t test, chi-square test, Fisher exact test, and Mann-Whitney U test was used to compare the groups according to data distribution. A *P* value of < 0.05 was considered to indicate statistical significance. Values are presented as mean ± SD or median (lower quartile, upper quartile) according to data distribution. Use of a multivariate logistic model including the main confounders to compare the risk of pregnancy complications with different endometrial preparation. Crude and adjusted odds ratios (ORs) with 95% confidence intervals (CIs) were calculated before and after adjusting for confounding variables including maternal age, infertility duration, cause of infertility, BMI, endometrial thickness and high-quality embryo transfer rate.

## Results

According to the above inclusion and exclusion criteria, a total 2333 singleton deliveries after frozen-thaw embryo transfer were enrolled in our study, including 550 cycles with programmed FET, 1783 cycles with tNC-FET. In further analysis according to BMI, group A included 1257 subjects, group B included 503 subjects and group C included 573 subjects.

### Background and treatment characteristics

There was no statistically significant difference in age, infertility duration, and number of embryo transfer. The mean maternal age was 31.70 and 31.49 years in the tNC-FET group and the programmed FET, respectively. Of women conceiving after tNC-FET, day 3 serum FSH level was significantly higher than programmed FET (6.40 ± 3.15 vs 5.95 ± 2.37, *P* = 0.002). But day 3 serum LH level was significantly higher in programmed FET than in the tNC-FET group (4.22 ± 2.50 vs 6.63 ± 4.61, *P* = 0.000). In the programmed FET group, more cycles were registered with tubal factor infertility compared with tNC-FET (36.1% vs 24.5%, *P* = 0.000), and more cycles were registered with male factor infertility in tNC-FET group compared with programmed FET group (16.9% vs 7.8%, *P* = 0.000). Further, more women in the programmed FET group were diagnosed with anovulatory infertility compared with tNC-FET (15.1% vs 4.0%, *P* = 0.000). ICSI was more often used in the tNC-FET groups than in programmed FET groups (45.5% vs 36.5%, *P* = 0.000), while IVF combined with ICSI was more often used in the programmed FET groups than in the tNC-FET groups (20.7% vs 9.30%, *P* = 0.000). Endometrial thickness of the tNC-FET group were significantly higher than in the programmed FET group (9.52 ± 1.66 vs 9.19 ± 1.52, *P* = 0.002). All background characteristics are shown in Table 1.

**Table 1:** Background and treatment characteristics of study participants according to endometrial preparation protocols

Parameter	tNC-FET group(*n* = 1783)	Programmed FET group(*n* = 550)	*P*
Age (years)	31.70 ± 4.10	30.49 ± 3.76	0.000
Infertility duration (years)	4.30 ± 2.77	4.22 ± 2.57	0.570
BMI (kg/m2)	23.05 ± 3.05	23.09 ± 3.17	0.538
Basal FSH(mIU/L)	6.40 ± 3.15	5.95 ± 2.37	0.002
Basal LH(mIU/L)	4.22 ± 2.50	6.63 ± 4.61	0.000
Cause of infertility, n (%)			
Tubal factor	36.1%(643/1783)	24.5%(135/550)	0.000
Anovulation	4.0%(71/1783)	15.1%(83/550)	0.000
Diminished ovarian reserve	1.9%(33/1783)	0.9%(5/550)	0.127
Endometriosis	1.4%(25/1783)	0.7%(4/550)	0.304
Unexplained infertility	4.8%(85/1783)	3.1%(17/550)	0.093
Male factor	16.9%(302/1783)	7.8%(43/550)	0.000
Other	35.0%(624/1783)	47.8%(263/550)	0.000
ART method, n (%)			
IVF	45.3%(807/1783)	42.7%(235/550)	0.296
ICSI	45.5%(811/1783)	36.5%(201/550)	0.000
IVF + ICSI	9.30%(165/1783)	20.7%(114/550)	0.000
Endometrial thickness (mm)	9.52 ± 1.66	9.19 ± 1.52	0.002
No.embroy transfer	1.81 ± 0.44	1.76 ± 0.44	0.570

### Obstetric and perinatal outcomes after programmed FET versus tNC-FET

Table 2 presents the results of both the crude analyses and the analyses adjusted for maternal age, infertility duration, cause of infertility, BMI, endometrial thickness and high-quality embryo transfer rate. There were no significant differences in the placenta previa, GDM, PPROM, and macrosomia between the tNC-FET and programmed FET groups (*P* > 0.05). The programmed FET cycles were associated to a higher risk of pregnancy-induced hypertension (PIH) (7.3% vs 4.4%, crude OR 1.71[1.16–2.54]; adjusted OR 1.845[1.03–3.30]), preterm birth (9.3% vs 12.0%, crude OR 1.33[0.98–1.80]; adjusted OR 1.71 [1.03–3.30]), low birth weight (5.3% vs 8.2%, crude OR 1.58[1.10–2.29]; adjusted OR 1.99[1.18–3.35]) compared with the tNC-FET cycles. CS was no longer significant in analyses after adjusting maternal age, infertility duration, cause of infertility, BMI, endometrial thickness and high-quality embryo transfer rate.

**Table 2:** Obstetric and perinatal outcomes after programmed FET group versus tNC-FET group

Characteristic	Treatment		Programmed FET vs. tNC-FET	
	tNC-FET(*n* = 1783)	Programmed FET(*n* = 550)	Crude OR (95% CI)	Adjusted OR (95% CI)
PIH	4.4%(78/1783)	7.3%(40/550)	1.71(1.16–2.54)^*^	1.845(1.03–3.30)^*^
Placenta previa	1.0%(17/1783)	0.9%(5/550)	0.95(0.35–2.60)	1.340(0.39–4.59)
GDM	9.4%(168/1783)	7.3%(40/550)	0.75(0.53–1.08)	0.901(0.56–1.47)
PPROM	2.5%(44/1783)	2.0%(11/550)	0.81(0.41–1.57)	1.119(0.46–2.70)
Cesarean section	68.0%(1212/1783)	72.9%(401/550)	1.27(1.03–1.57)^*^	1.026(0.77–1.36)
Preterm birth	9.3%(166/1783)	12.0%(66/550)	1.33(0.98–1.80)	1.714(1.11–2.66)^*^
Low birth weight (< 2500 g)	5.3%(95/1783)	8.2%(45/550)	1.58(1.10–2.29)^*^	1.988(1.18–3.35)^*^
Macrosomia (> 4500 g)	9.9%(177/1783)	11.6%(64/550)	1.10(0.88–1.62)	1.033(0.66–1.61)

^*^P < 0.05 (adjusted for maternal age, infertility duration, cause of infertility, BMI, endometrial thickness and high-quality embryo transfer rate)

### Subanalyses of different BMI patients on obstetric and perinatal outcomes after programmed FET versus tNC-FET

**Fig. 1:**
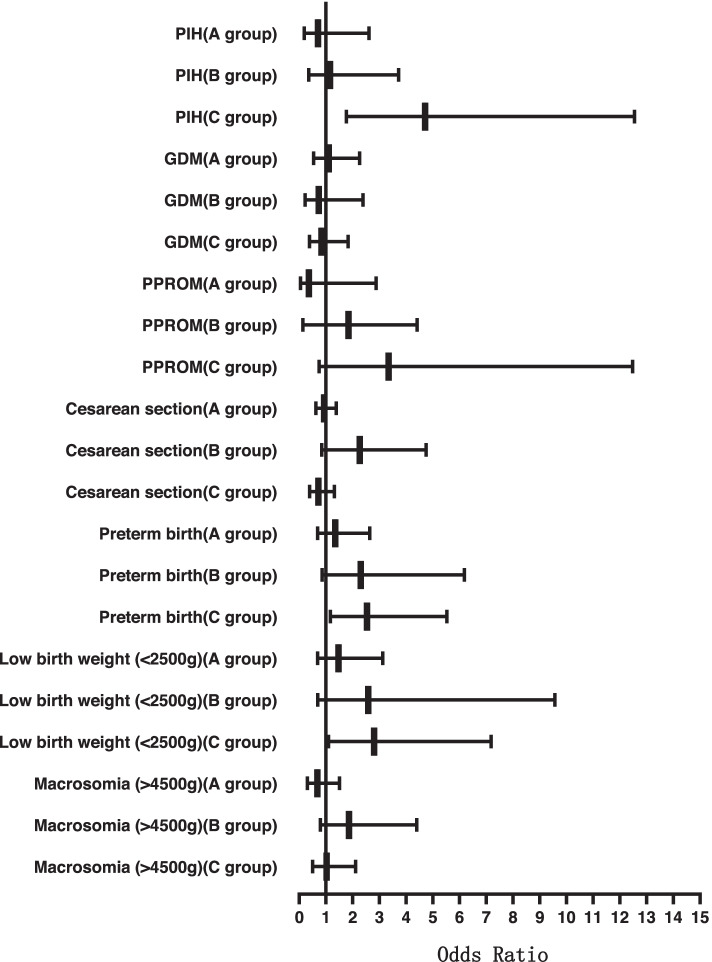
Odds ratios of obstetric and perinatal outcomes among different BMI patients with different endometrial preparation protocols

After dividing the patients into three groups according to the BMI, the comparison results of pregnancy complications and birth weights among the three groups of patients with different endometrial preparation programs are presented in Table 3. The programmed FET cycles were associated to a higher risk of PIH in group C (14.4% vs 6.2%, crude OR 2.55 [1.42–4.55]; adjusted OR 4.71 [1.77–12.55]) compared with the tNC-FET cycles. But there was no statistically significant difference in group A and group B. Moreover, there was no an association between endometrial preparation protocols and risk of preterm birth (crude OR 1.24 [0.72–2.12]) and low birth weight (crude OR 1.93 [0.98–3.81]) in group C before correction for confounding factors. When the confounding variables including maternal age, infertility duration, cause of infertility, endometrial thickness and high-quality embryo transfer rate were added to the logistic regression models, the programmed FET cycles were associated to a higher risk of preterm birth (12.8% vs 10.6%, adjusted OR 2.54 [1.16–5.53]) and low birth weight (9.1% vs 4.9%, adjusted OR 2.81 [1.16–5.53]) in group C compared with the tNC-FET cycles. There also was no statistically significant difference in group A and group B. It can be seen from Fig. 1 that with the body mass index increase, programmed FET group compared with the tNC-FET group, the risk of PIH, preterm birth and low birth weight also increases. There was no statistically significant difference in placenta previa, GDM, CS, PPROM and macrosomia (*P*>0.05).

**Table 3:** Subanalyses of different BMI patients on obstetric and perinatal outcomes after programmed FET versus tNC-FET

Parameter	tNC-FET	Programmed FET	Programmed FET vs. tNC-FET	Adjusted OR (95% CI)
	%(n/)	%(n/)	Crude OR (95% CI)	
PIH				
A(*n* = 1257)	2.7%(27/1010)	2.4%(6/247)	0.91(0.37–2.22)	0.70(0.19–2.61)
B(*n* = 503)	7.0%(27/387)	6.0%(7/116)	0.86(0.36–2.02)	1.15(0.36–3.72)
C(*n* = 573)	6.2%(24/386)	14.4%(27/187)	2.55(1.424–4.548)^*^	4.712(1.77–12.54)^*^
Placenta previa				
A(*n* = 1257)	0.9%(9/1010)	1.2%(3/247)	1.37(0.37–5.09)	1.298(0.24–7.10)
B(*n* = 503)	1.3%(5/387)	1.7%(2/116)	1.34(0.26–7.00)	–
C(*n* = 573)	0.8%(3/386)	0.0%(0/187)	–	–
GDM				
A(*n* = 1257)	7.5%(76/1010)	6.5%(16/247)	0.85(0.49–1.49)	1.107(0.54–2.26)
B(*n* = 503)	9.8%(38/387)	6.0%(7/116)	0.59(0.26–1.36)	0.731(0.22–2.39)
C(*n* = 573)	14.0%(54/386)	9.1%(17/187)	0.62(0.35–1.09)	0.838(0.38–1.83)
PPROM				
A(*n* = 1257)	2.6%(26/1010)	1.2%(3/247)	0.47(0.14–1.55)	0.365(0.05–2.88)
B(*n* = 503)	1.8%(7/387)	1.7%(2/116)	0.95(0.20–4.65)	1.845(0.14–4.41)
C(*n* = 573)	2.8%(11/386)	3.2%(6/187)	1.13(0.41–3.10)	3.345(0.74–12.48)
Cesarean section				
A(*n* = 1257)	63.3%(639/1010)	64.4%(159/247)	1.05(0.79–1.40)	0.932(0.63–1.38)
B(*n* = 503)	71.1%(275/387)	80.2%(93/116)	1.65(0.99–2.73)	2.268(0.84–4.75)
C(*n* = 573)	77.2%(298/386)	79.7%(149/187)	1.16(0.76–1.78)	0.717(0.39–1.31)
Preterm birth				
A(*n* = 1257)	8.5%(86/1010)	11.3%(28/247)	1.37(0.88–2.16)	1.35(0.69–2.64)
B(*n* = 503)	10.1%(39/387)	12.1%(14/116)	1.23(0.64–2.34)	2.31(0.86–6.18)
C(*n* = 573)	10.6%(41/386)	12.8%(24/187)	1.24(0.72–2.12)	2.54(1.16–5.53)^*^
Low birth weight (< 2500 g)				
A(*n* = 1257)	5.3%(54/1010)	8.9%(22/247)	1.73(1.03–2.90)^*^	1.467(0.69–3.13)
B(*n* = 503)	5.7%(22/387)	5.2%(6/116)	0.901(0.36–2.29)	2.585(0.70–9,57)
C(*n* = 573)	4.9%(19/386)	9.1%(17/187)	1.93(0.98–3.81)	2.806(1.10–7.18)^*^
Macrosomia (> 4500 g)				
A(*n* = 1257)	8.0%(81/1010)	6.1%(15/247)	0.74(0.41–1.31)	0.68(0.31–1.51)
B(*n* = 503)	11.4%(44/387)	18.1%(21/116)	1.72(0.98–3.04)	1.86(0.79–4.40)
C(*n* = 573)	13.5%(52/386)	15.0%(28/187)	1.13(0.69–1.86)	1.03(0.50–2.11)

^*^P < 0.05 (adjusted for maternal age, infertility duration, cause of infertility, endometrial thickness and high-quality embryo transfer rate); − None data because of the small amount of subjects

## Discussion

Our results of adverse obstetric and perinatal outcomes after procedural FET are similar to those of some recently published studies [10–12, 14, 15]. In our study we were able to adjust for maternal age, infertility duration, cause of infertility, BMI, endometrial thickness and high-quality embryo transfer rate. And to reduce the influence of confounding factors, only singletons were included. In the multiple logistic regression analysis, we illustrated that the risk of PIH, preterm birth and low birth weight was higher after FET protocols with no corpus luteum (programmed FET) compared with pregnancies after FET protocols creating a corpus luteum (tNC-FET). Moreover, grouped by the BMI of patients, programmed FET group compared with the tNC-FET group, we showed a tendency toward increasing risk of PIH, preterm birth and low birth weight as the weight of the patient increases.

A large epidemiologic study by Saito et al [10] in Japan examined the risk for HPD in autologous pregnancies regarding the FET protocols used natural cycle (*n* = 29,760) and programmed cycle (*n* = 75,474). They determined that compared with a natural-cycle FET, pregnancies after programmed FET had increased odds of HPD (adjusted OR, 95% CI 1.43[1.14–1.80]). In our study, the risk of PIH (adjusted OR, 95% CI 1.845 [1.031–3.300]) was also increased in programmed FET group compared with the tNC-FET group. Otherwise, we also showed a tendency toward increasing risk of preterm birth and low birth weight, it may be related to increased risk of PIH After we excluded PIH in the regression analysis, the increased risk of preterm birth and low birth weight was not present (Supplementary Table 1).

Estrogen and progesterone are essential for the development of a normal placenta during pregnancy. Altered levels of these sex steroid hormones may lead to placenta-related complications in programmed FET cycle [16]. Due to failure of normal decidualization and excessive invasion of trophoblasts, low progesterone levels in early pregnancy may lead to placental implantation [17]. In contrast, some studies have shown that later development of pre-eclampsia is associated with high progesterone in early third trimester [18]. A recent overview has been shown that FET cycles have been associated with an increased risk for adverse obstetric and perinatal outcomes of a pregnancy, the absence of the CL and the following deficient circulatory adaptations during early pregnancy in programmed cycles may play a role in these increased risk [6]. The CL is an important source of reproductive hormones before the establishment of the placenta as a source of pregnancy maintaining reproductive hormones, such as progesterone and estrogen. Moreover, CL also produces vasoactive products such as relaxin, vascular endothelial growth factor (VEGF), and angiogenic metabolites of estrogen [19]. They are also key factors in pregnancy maintenance. The vasoactive products produced by CL are thought to play an important role in early placenta formation and maternal circulation adaptation, and abnormal early placenta is often considered to be a key step in the development of preeclampsia [20].

BMI also have important influence in IVF/ICSI outcomes. The adverse effects of overweight/obesity on pregnancy outcomes have been widely confirmed, including dysregulation of the hypothalamic-pituitary-ovarian axis, ovulation disorders, impaired preimplantation embryo, and higher risk of miscarriage [21]. Meanwhile, pre-pregnancy overweight and obesity are related to many adverse obstetric and perinatal outcomes of a pregnancy, including HPD, GDM, fetal macrosomia, fetal structural anomalies, CS, preterm delivery and low neonatal Apgar score [22–25]. A recent study in Korean women demonstrated that pre-pregnancy overweight and obesity are more closely related to the adverse obstetric outcomes than excess weight gain during pregnancy [24]. In our study, considering the important impact of BMI on obstetric and perinatal outcomes of a pregnancy, we further grouped patients according to their pre-pregnancy BMI. We demonstrate that the risk of PIH in obese patients was increased in programmed FET group compared with the tNC-FET group. And a tendency toward increasing risk of PIH as the weight of the patient increases was observed. The increased risk of preterm and low birth weight has also been observed, and through further analysis we believe that it is related to the increased risk of PIH (Supplementary Table 2). PCOS/anovulation also need to be focused. In the Swedish study [14], the investigators adjusted for anovulation, and the Chinese study included only normoovulatory women [11] both found a higher risk of PIH. We were not able to adjust for PCOS/anovulation; however, analyses excluding all women with PCOS/anovulation did not change our results (Supplementary Table 3).

An increased risk of macrosomia in the programmed FET group was also found in the Swedish study [14]. Reasons for the increased risk of high birth weight in pregnancy after FET include maternal factors [26, 27], embryo culture media [28, 29], embryo transfer status (cleavage stage/blastocyst) [3, 30], and the quality of transferred embryos during FET. Our study also shows a tendency toward increasing risk of macrosomia. Recent study also showed that the freezing/thawing process (both slow freeze and verification) may cause epigenetic changes, which may be related to newborn weight gain [31, 32]. In addition, some studies have shown that the risk of adverse obstetric and perinatal outcomes of a pregnancy such as CS increases after in the programmed FET group [15, 33]. In our study, the risk of cesarean section dose not increase in the programmed FET group significantly, but the rate of CS has showed higher in programmed FET group (72.9%) than tNC-FET group (68%). The higher risk of adverse obstetric and perinatal outcomes after the programmed FET treatment may be responsible for the higher risk of CS.

A major strength of this study is the complete birth cohort of singletons conceived after FET in China, which minimizes the risk of selection bias. In the multiple logistic regression analysis, we were able to adjust for maternal age, infertility duration, cause of infertility, BMI, endometrial thickness and high-quality embryo transfer rate. Considering the influence of BMI on obstetric and perinatal outcomes of a pregnancy, we further grouped according to BMI, firstly. Women treated with hCG trigger during FET cycle was not included in our study, because the key information absence, which is a main limitation of our study. The further study about programmed FET mNC-FET and tNC-FET should be performed in Chinese population.

## Conclusion

We showed a tendency toward increasing risk of PIH in Programmed FET cycle compared with the tNC-FET cycle, and the risk of PIH increases as BMI increases. Increased risk of preterm birth and low birth weight is linked to increased risk of PIH.

## Supplementary Information

**Additional file 1: Supplementary Table 1.** Excluded PIH subjects in the regression analysis, the increased risk of preterm birth and low birth weight was not present. We believe that it is related to the increased risk of PIH. **Supplementary Table 2.** Excluded PIH subjects in the regression analysis, the increased risk of preterm birth and low birth weight was not present in three groups. We believe that it is related to the increased risk of PIH. **Supplementary Table 3.** Excluding all women with PCOS/anovulation in the regression analysis, the risk of PIH (adjusted OR, 95% CI 4.62 [1.72–12.45]) was also increased in programmed FET group compared with the tNC-FET group in group C.

## Data Availability

The data used or analyzed during the current study are included within the article. The datasets are not publicly available due to the hospital policy and personal privacy. However, the datasets are available from the corresponding author on reasonable request.

## Abbreviations

BMI: Body mass index
CI: Confidence intervals
CL: Corpus luteum
CS: Cesarean section
e-SET: Elective single-embryo transfer
FET: Frozen embryo transfer
GDM: Gestational diabetes mellitus
hCG: Human chorionic gonadotropin
IVF/ICSI: In vitro fertilization/intracytoplasmic sperm injection
mNC-FET: Modified natural cycle
OHSS: Ovarian hyperstimulation syndrome
OR: Odds ratios
PGT: Preimplantation genetic testing
PIH: Pregnancy-induced hypertension
PPROM: Preterm premature rupture of membranes
PTB: Preterm birth
tNC-FET: True natural cycle FET
