# Supplementary material for: Adverse obstetric and perinatal outcomes in 2333 singleton pregnancies conceived after different endometrial preparation protocols: a retrospective study in China
Source: BMC Pregnancy Childbirth. 2022 May 1;22:378. doi: 10.1186/s12884-022-04682-3 (PMC9063113; doi:10.1186/s12884-022-04682-3)
Supplement: Supplementary file 1 — Additional file 1: Supplementary Table 1. Excluded PIH subjects in the regression analysis, the increased risk of preterm birth and low birth weight was not present. We believe that it is related to the increased risk of PIH. Supplementary Table 2. Excluded PIH subjects in the regression analysis, the increased risk of preterm birth and low birth weight was not present in three groups. We believe that it is related to the increased risk of PIH. Supplementary Table 3. Excluding all women with PCOS/anovulation in the regression analysis, the risk of PIH (adjusted OR, 95% CI 4.62 [1.72–12.45]) was also increased in programmed FET group compared with the tNC-FET group in group C. [file 12884_2022_4682_MOESM1_ESM.docx]

**Supplement**

**Supplementary Table 1**

Excluded PIH subjects in the regression analysis, the increased risk of preterm birth and low birth weight was not present. We believe that it is related to the increased risk of PIH.

| Characteristic | Treatment | | Programmed FET vs. tNC-FET | |
| --- | --- | --- | --- | --- |
|  | tNC-FET(n=1783) | Programmed FET(n=550) | Crude OR (95% CI) | Adjusted ORa (95% CI) |
| Preterm birth | 8.4%（143/1705） | 10.6%（54/510） | 1.46（0.97-2.20） | 1.40（0.85-2.28） |
| Low birth weight (<2500g) | 4.8%（82/1705） | 6.9%（35/510） | 1.29(0.93-1.80) | 1.99（1.18-3.35） |

(adjusted for maternal age, infertility duration, cause of infertility, endometrial thickness and high-quality embryo transfer rate)

**Supplementary Table 2**

Excluded PIH subjects in the regression analysis, the increased risk of preterm birth and low birth weight was not present in three groups. We believe that it is related to the increased risk of PIH.

| Parameter | tNC-FET | Programmed FET | Programmed FET vs. tNC-FET | |
| --- | --- | --- | --- | --- |
|  | %(n/) | %(n/) | Crude OR (95% CI) | Adjusted ORa (95% CI) |
| Preterm birth |  |  |  |  |
| A（n=1257) | 7.9%(78/983) | 10.8%(26/241) | 1.40(0.88-2.24) | 1.22（0.60-2.48） |
| B(n=503) | 8.6%(31/360) | 11.0%(12/109) | 1.31(0.65-2.65) | 2.41（0.76-7.58） |
| C(n=573) | 9.4%(34/362) | 10.0%(16/160) | 1.07(0.57-2.00) | 1.56（0.63-3.84） |
| Low birth weight (<2500g) |  |  |  |  |
| A（n=1257) | 5.1%(50/983) | 8.3%(20/241) | 1.69（0.99-2.90） | 1.24（0.55-2.79） |
| B(n=503) | 4.4%(16/360) | 4.6%(5/109) | 1.03（0.37-2.89） | 3.46（0.64-18.57） |
| C(n=573) | 4.4%(16/362) | 6.3%(10/160) | 1.44（0.64-3.25） | 1.39（0.45-4.28） |

(adjusted for maternal age, infertility duration, cause of infertility, endometrial thickness and high-quality embryo transfer rate)

**Supplementary Table 3**

Excluding all women with PCOS/anovulation in the regression analysis, the risk of PIH (adjusted OR, 95% CI 4.62 [1.72-12.45]) was also increased in programmed FET group compared with the tNC-FET group in group C.

| Parameter | tNC-FET | Programmed FET | Programmed FET vs. tNC-FET | |
| --- | --- | --- | --- | --- |
|  | %(n/) | %(n/) | Crude OR (95% CI) | Adjusted ORa (95% CI) |
| PIH |  |  |  |  |
| A（n=1257) | 8.6%(84/975) | 11.5%(25/218) | 0.86（0.33-2.26） | 0.70（0.19-2.61） |
| B(n=503) | 10.0%(37/371) | 12.5%(12/96) | 0.96（0.38-2.43） | 1.59（0.47-5.37） |
| C(n=573) | 10.9%(40/366) | 15.7%(24/153) | 2.64（1.43-4.87）***** | 4.62（1.72-12.45）***** |
| Preterm birth |  |  |  |  |
| A（n=1257) | 8.0%(79/949) | 10.8%(23/213) | 1.39(0.85-2.28) | 1.16（0.56-2.41） |
| B(n=503) | 8.4%(29/347) | 11.1%(10/90) | 1.37(0.64-2.93) | 1.09（0.95-1.25） |
| C(n=573) | 9.6%(33/343) | 12.3%(16/130) | 1.32(0.70-2.49) | 1.56（0.63-3.84） |
| Low birth weight (<2500g) |  |  |  |  |
| A（n=1257) | 5.1%(49/949) | 8.9%(19/213) | 1.84（1.06-3.20） | 1.35（0.60-3.03） |
| B(n=503) | 4.6%(16/347) | 5.6%(5/90) | 1.22（0.43-3.41） | 3.46（0.64-18.57） |
| C(n=573) | 4.1%(14/343) | 6.9%(9/130) | 1.75（0.74-4.14） | 1.78（0.57-5.56） |

**^*^***P*<0.05 (adjusted for maternal age, infertility duration, cause of infertility, endometrial thickness and high-quality embryo transfer rate)
